# Physico-Chemical Properties and Push-Out Bond Strength to Root Dentine of Calcium Silicate-Based Sealers

**DOI:** 10.3390/jfb16040131

**Published:** 2025-04-03

**Authors:** Ivana Milanovic, Vesna Miletic, Bojan Dzeletovic, Djordje Antonijevic, Tatjana Savic Stankovic, Danilo Pavlovic, Ana Despotovic, Violeta Petrovic

**Affiliations:** 1 DentalNet Research Group, School of Dental Medicine, University of Belgrade, Rankeova 4, 11000 Belgrade, Serbia; ivana.milanovic@stomf.bg.ac.rs (I.M.); bojan.dzeletovic@stomf.bg.ac.rs (B.D.); tanja.savic@stomf.bg.ac.rs (T.S.S.); danilo.pavlovic@stomf.bg.ac.rs (D.P.); 2 Faculty of Medicine and Health, Sydney Dental School, University of Sydney, Surry Hills, NSW 2010, Australia; vesna.miletic@sydney.edu.au; 3 Department of Anatomy, School of Dental Medicine, University of Belgrade, Dr Subotica 8, 11000 Belgrade, Serbia; djordje.antonijevic@stomf.bg.ac.rs; 4 Institute for Biological Research “Siniša Stanković”—National Institute of the Republic of Serbia, University of Belgrade, Bulevar despota Stefana 142, 11000 Belgrade, Serbia; ana.despotovic@ibiss.bg.ac.rs

**Keywords:** calcium silicate, endodontic sealers, alkalinity, radiopacity, flexural strength, dislocation resistance

## Abstract

The calcium silicate-based sealers currently available on the market have different compositions and formulations, which is why their physical and chemical properties may vary. (1) The aim of the study was to measure the physico-chemical properties of calcium silicate-based sealers and their push-out bond strength to root dentine, comparing two push-out testing protocols. (2) Standardized specimens of EndoSequence BC, BioRoot RCS, MTA Fillapex, and AH Plus (control) were subjected to pH measurements over 28 days. Radiopacity was measured using a CCD sensor, and flexural strength was assessed using a three-point bending setup. Push-out bond strength was measured in coronal, middle, and apical sections of 40 single-root teeth (conventional method), and cylindrical cavities were prepared for all sealers on the same root dentine disks in 11 third molars (disk method). (3) EndoSequence BC exhibited a higher pH than MTA Fillapex and the highest radiopacity (*p* < 0.05). The highest flexural and push-out bond strengths were found for AH Plus. The push-out bond strength of EndoSequence BC and BioRoot RCS was higher than MTA Fillapex (*p* < 0.05). The conventional and disk methods exhibited similar push-out bond strength results, but the data were more homogeneously distributed in the disk method. (4) All calcium silicate-based sealers exhibited a higher pH than AH Plus. MTA Fillapex did not meet the ISO standard. Calcium silicate-based sealers showed weaker performance in terms of physical properties compared to AH Plus.

## 1. Introduction

Current hydraulic calcium silicate-based sealers are available as powder/liquid systems or pre-mixed ready-to-use syringes. The main difference between the two forms is the way the materials obtain water necessary for hydration and setting [1]. In powder/liquid-based sealers, such as BioRoot RCS (Septodont, Saint Maur Des Fosses, France), hydration is initiated before insertion into the root canal. In pre-mixed ready-to-use sealers, such as EndoSequence BC (Brassler, Savannah, GA, USA), residual moisture inside the root canal and dentine humidity provide water necessary for the hydration and setting reaction [2].

Calcium silicate-based sealers are indicated in combination with monocone gutta-percha [3]. A greater amount of sealer inside the root canal increases the importance of material testing in terms of physico-chemical properties and bonding efficiency to root canal dentine, especially considering the differences in material composition with a potential effect on hydration.

Bonding efficiency is universally measured using push-out bond strength test. Specimen preparation may differ, e.g., a “conventional” method utilizes single-rooted teeth with the original root canal space prepared and obturated whilst a “disk” method utilizes artificial cylindrical cavities prepared for all tested sealers on the same root dentine disk [4]. The conventional method uses original root canal anatomy but requires a substantial number of teeth and does not account for differences in substrate (dentine age, tertiary dentine formation, elasticity, frequency and diameter or dentinal tubules). The disk method allows better standardization, eliminates dentine differences and reduces the required number of teeth.

Previous studies compared bonding efficiency of calcium silicate-based sealers combined with gutta-percha using the conventional method [5,6,7,8]. Nagas et al. found lower bond strength values for AH Plus compared to iRoot SP [5], while AH Plus and BC performed similarly in another study [6]. Although the approaches in these studies are close to a clinical situation, gutta-percha is easily deformed, which may impact load distribution and lead to inaccurate plunger positioning. Therefore, bonding efficiency is more accurately determined when root canal space is filled only with sealer, without gutta-percha. More recent studies have utilized the disk method and sealer-only approach to test the bonding efficiency of calcium silicate-based sealers [9,10]. There are no findings in the literature that compare the results of two push-out models for testing sealer bonding to root dentine.

High alkalinity of calcium silicate-based sealers is associated with their antimicrobial activity [11] and apatite nucleation potentially promoting bone regeneration [12]. The pH of calcium silicate-based sealers was previously shown to be in the range of 9–12 over different time periods [13,14,15]. BioRoot RSC exhibited high alkaline values after being immersed in distilled water for 24 h and maintained these values even after six months [13,15]. In contrast, MTA Fillapex reached a pH of 8.0 after 24 h, which then increased to 8.8 after six months [13,15]. AH Plus, however, maintained pH values below 7 throughout all measured intervals over a 21-day period [14].

Radiopacity of calcium silicate-based sealers is increasingly based on alternative radiopacifiers, other than bismuth oxide, previously used in calcium silicate cements, due to effect of bismuth on tooth discoloration [16,17,18]. Zirconium oxide is used in most calcium silicate-based sealers, as it does not affect hydration and tooth color [19]. Radiopacity values of AH Plus vary from 11.8 [20] to 6.85 [21]. MTA Fillapex and BioRoot RCS demonstrated radiopacity values between 5 and 7 [20,21]. Candeiro et al. reported the radiopacity for Endosequence BC to be 3.83, which is a little above the ISO standard [22]. These inconsistent data from the literature are most likely caused by the use of different measuring techniques [20,21,22].

The mechanical properties of calcium silicate-based sealers have not been extensively studied despite the need to strengthen endodontically treated teeth or withstand forces during post-space preparation after root canal obturation. Smear layer removal using maleic acid or EDTA may adversely affect the mechanical properties and microstructure of MTA [23]. Simulated body fluids and bacterial esterases were found to affect mechanical properties such as microhardness and compressive strength of a calcium silicate-based sealer (EndoSequence BC), as well as other resin-based sealer types [24]. No data were found in the literature for the flexural strength of calcium silicate-based sealers.

The aim of this study was to measure push-out bond strength to root dentine of calcium silicate-based sealers comparing two push-out testing protocols, as well as physico-chemical properties, such as pH, radiopacity, and flexural strength (Fs). The null hypotheses were as follows. (1) There is no statistically significant difference in push-out bond strength between tested sealers, (2) there is no statistically significant difference in push-out bond strength determined by the conventional and “disk” method and (3) there are no statistically significant differences in pH, radiopacity, and flexural strength among tested materials.

## 2. Materials and Methods

### 2.1. Materials

The study protocol and use of extracted teeth for research purposes were approved by the School Ethics Committee (No. 36/15, 21 June 2016). Table 1 presents information on the sealers included in the present study. All specimens in the following experiments were prepared by one operator (experienced clinician), while the measurements were performed by another operator to minimize the operator bias. The blinding procedure was implemented by coding and randomizing the samples.

### 2.2. Specimen Preparation for Push-Out Bond Strength Measurement

#### 2.2.1. Conventional Method

Forty human single-rooted teeth extracted for periodontal or endodontic reasons were cleaned of debris and stored in 0.2% thymol refrigerated at 4 °C for no longer than 6 months post-extraction. The root length of the selected teeth was 13 ± 1 mm. Teeth were embedded in acrylic (Duracryl plus, Spofa dental, KavoKerr, Brea, CA, USA) in standardized silicon molds 10 × 10 × 15 mm up to the cemento-enamel junction. The crowns were cut off using a diamond saw at 0.7 mm speed under coolant (Isomet Buehler, Lake Bluff, IL, USA). Three 1 ± 0.1 mm disks were cut from each tooth perpendicular to the long axis. Disk thickness was verified with a digital caliper (d = 0.01 mm). The first disk was sectioned 3 mm coronally to root apex and the subsequent two disks were sectioned 3 mm coronally to the previous section. In this manner, 3 specimens were obtained from each tooth: coronal, middle and apical, resulting in 120 specimens in total.

The original root canal space of each specimen was prepared using a fissure diamond bur 1.2 mm in diameter (Dentsply/Maillefer, Ballaigues, Switzerland) in a fixed handpiece to produce standardized cavities. The disks were then subsequently immersed in the following solutions: 2% NaOCl, 17% EDTA and distilled water for 60 s each. The disks were blot-dried and randomly allocated to each sealer group (*n* = 10/group).

Each disk was filled with a respective sealer, previously mixed according to manufacturer’s instructions and inserted using a probe in a vibrating motion. Excess material was removed using a plastic instrument. The specimens were wrapped in gauze previously immersed in Hank’s balanced salt solution and kept in an incubator at 37 °C for 7 days.

#### 2.2.2. Disk Method

Eleven human maxillary third molars, extracted for orthodontic reasons, were cleaned of debris and stored in 0.2% thymol refrigerated at 4 °C for no longer than 6 months. Teeth were embedded in acrylic (Duracryl plus, Spofa dental, KavoKerr, CA, USA) in standardized silicon molds 10 × 10 × 15 mm up to the cemento-enamel junction. The crowns were cut off at the cemento-enamel junction using a diamond saw at 0.7 mm speed under coolant perpendicular to the long axis. One 1 ± 0.1 mm disk was sectioned from the middle segment of each tooth. In each disk, 4 standardized cavities, 1.2 mm in diameter, were prepared using a fissure diamond bur 1.2 mm in diameter (Dentsply/Maillefer, Ballaigues, Switzerland) in a fixed handpiece to produce standardized cavities. The disks were then subsequently immersed in the following solutions: 2% NaOCl, 17% EDTA and distilled water for 60 s each and then blot-dried.

Cavities in each disk were randomly allocated to each group and then filled with the respective sealer, previously mixed according to manufacturer’s instructions using a probe in a vibrating motion. Excess material was removed using a plastic instrument. The specimens were wrapped in gauze previously immersed in Hank’s balanced salt solution and kept in an incubator at 37 °C for 7 days. In this way, each disk in the disk method contained all 4 sealers.

### 2.3. Measurement of Push-Out Bond Strength

Push-out bond strength to root dentine of each tested sealer was performed using a universal testing machine (PCE-FM 200) by placing each disk between two supports, so that the sealer dislocation is not blocked by the supports. A cylindrical, custom-made stainless-steel indenter 0.8 mm in diameter was used to apply force to the sealer at 1 mm/min speed until the sealer dislocated from the root canal space. Bond strength σ/MPa was calculated using the following formula:

σ (Mpa) = F/2rπh(1)

where F/N is the maximum load measured at fracture, r is cavity radius (0.6 mm) and h is specimen height (1 mm).

### 2.4. Specimen Preparation and Measurement of pH and Radiopacity

For pH measurements, power and sample size analyses were performed in G*Power version 3.1 (www.psychologie.hhu.de) for “Means—Many groups ANOVA: repeated measurements, within- between interaction”. The required sample size per group to detect a medium effect size f = 0.5 with alpha 0.05, power 0.9, (number of groups 4, number of measurements 6, total sample size 12) resulted in 3 specimens per group.

Standardized specimens (*n* = 3/group/per experiment) were prepared in silicon molds, 5 mm in diameter and 2 mm thick, by mixing the sealer according to the manufacturer’s instructions and filling each mold using a probe in a vibrating motion to prevent air entrapment. Excess material was removed using a plastic instrument. Specimens for pH were allowed to initially set for 30 min prior to pH measurements. Specimens for radiopacity were wrapped in gauze previously immersed in Hank’s balanced salt solution and allowed to completely set for 7 days in an incubator at 37 °C prior to radiopacity measurements.

Each specimen was placed in a glass vial containing 10 mL deionized water and stored at 37 °C, 100% humidity. The pH assessment was performed after 1, 3, 7, 14, 21 and 28 day(s) of immersion using a pH meter (pH-vision Microcomputer 6071, JENCO Electronics Ltd., Linkou Shiang, Taiwan) equipped with a pH electrode (Hanna Instruments WTW GmbH, Woonsockets, RI, USA). Prior to each measurement cycle, the pH electrode was calibrated using two calibration solutions pH 7 and 10 (Reagecon Diagnostics Ltd., Clare, Ireland). Each measurement was performed in triplicate and a mean value was calculated. After each measurement, the specimens were immersed in fresh deionized water and kept at 37 °C.

Radiopacity measurements were performed in accordance with the ISO 6876:2012 standard using a digital CCD intraoral x-ray sensor (CCD sensor, Trophy, Saint Maur, France) with the following parameters: 0.07 s exposure, 70 kV voltage, 10 mA current and a tube-to-specimen distance of 35 cm [25]. Each specimen was radiographed with a 99.6% pure aluminum standard made of 10 Al plates with 1 mm increasing thickness. Digital x-rays were kept as TIFF files and analyzed using a histogram tool in Adobe Photoshop CS7 (Adobe Systems, San Jose, CA, USA) [26]. Each specimen was analyzed in triplicate and a mean value of the grayscale tone was calculated. A calibration curve graph was created using these mean values and Al thickness logarithm, which allowed conversion of the grayscale tone to radiopacity expressed as Al thickness.

### 2.5. Specimen Preparation and Measurement of Flexural Strength

For Fs measurements “ANOVA: Fixed effects, omnibus, one-way”, the required sample size per group to detect a large effect size f = 0.8 with alpha 0.05, power 0.9, (number of groups 4, total sample size 28) resulted in 7 samples per group. Stick-shaped specimens (*n* = 8/group) were prepared in standardized 10 × 2 × 2 mm silicon molds. Sealers were mixed according to the manufacturer’s instructions and placed in the molds using a probe in a vibrating motion to prevent air entrapment. Excess material was removed using a plastic instrument. The specimens were wrapped in gauze previously immersed in Hank’s balanced salt solution and kept in an incubator at 37 °C for 7 days.

Fs was performed using a universal testing machine (PCE-FM 200, PCE Instruments UK Ltd., Southampton, UK) in a three-point test setup, at 1 mm/min speed until fracture. Fs MPa was determined using the following equation:

Fs = 3Fl/(2bh^2)(2)

where F/N is the maximum load measured at fracture, l/mm is the distance between supports, b/mm is sample width, and h/mm is sample height.

### 2.6. Statistical Analysis

Data for push-out bond strength (conventional method) were tested using GLM for factors “sealer” and “location” with factor interaction included. Data for pH were analyzed using a general linear model (GLM) for factors “sealer” and “time” with factor interaction included. Data for radiopacity, Fs and push-out bond strength (disk method) were analyzed using one-way analysis of variance (ANOVA) with Tukey’s post hoc test. In case of significant interaction, further one-way ANOVA was used to analyze data within each factor. The level of significance was set at α = 0.05.

## 3. Results

**Figure 1:**
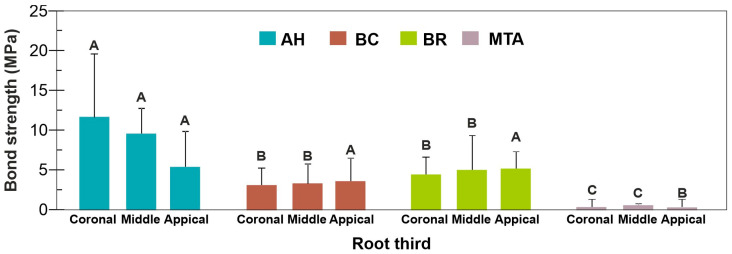
Bond strength (mean ± SD) to root dentine of the tested sealers measured using the conventional method. Within each location, sealers sharing the same letter are not significantly different (*p* > 0.05).

Push-out bond strength determined by the conventional method, two-way ANOVA for factors “sealer” and “location” showed a significant interaction of factors (*p* = 0.009). Significant differences were found within factor “sealer” (*p* < 0.05), whilst no significant difference was found within factor “location” (*p* = 0.094). In the subsequent one-way ANOVA, bond strength analysis within factor “sealer” revealed differences between tested sealers at coronal, middle and apical root third. In the coronal and middle third, AH showed significantly higher bond strength values than the calcium silicate-based sealers (*p* < 0.05). BC and BR showed no statistically significant differences (*p* > 0.05), whilst bond strength values for MTA were significantly lower than other sealers (*p* < 0.05). In the apical third, no significant differences in bond strength were found for AH, BC and BR (*p* > 0.05), but the values for MTA were again significantly lower than other sealers (*p* < 0.05) (Figure 1).

**Figure 2:**
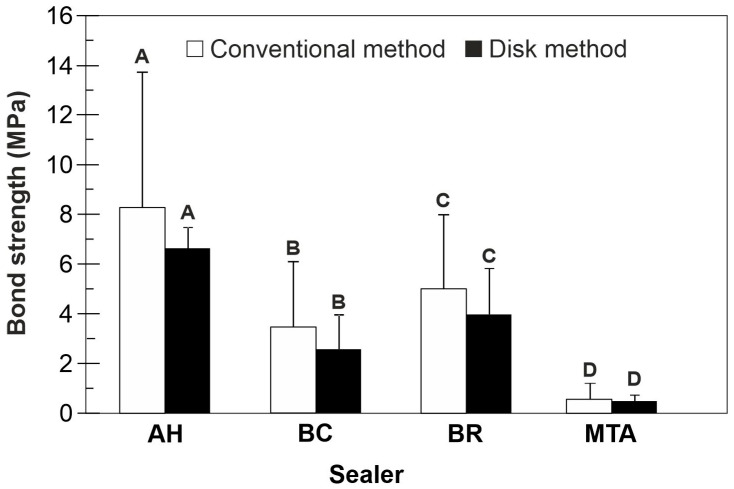
Bond strength values (mean ± SD) obtained by the conventional and disk method. Within each location, sealers sharing the same letter are not significantly different (*p* > 0.05).

Figure 2 shows the bond strength values obtained using the disk method and their relation to the mean bond strength values for each sealer obtained using the conventional method. The disk method showed significantly the highest bond strength values for AH and the lowest for MTA (*p* < 0.05). BR and BC resulted in comparable values (*p* > 0.05) between AH and MTA. When comparing the two methods, no significant differences in bond strength values were found in any sealer group (*p* > 0.05), albeit the disk method resulted in more homogenous, i.e., less dispersed values than the conventional method.

BC sealer exhibited significantly higher pH (11.05 ± 0.91) than MTA (10.01 ± 0.75) and AH (9.16 ± 0.61) (*p* < 0.05) and comparable pH to BR (10.49 ± 0.57) (*p* > 0.05). Calcium silicate-based sealers BC, BR and MTA showed significantly higher pH than AH control (*p* < 0.05). Significant interaction was found for factors “sealer” and “time” (*p* = 0.029). Subsequent analysis within each sealer showed that the pH of AH was consistent over time with no significant differences between time intervals (*p* > 0.05). Table 2 presents pH values for each sealer across test intervals and differences may be observed for calcium silicate-based sealers. BC and BR showed comparable pH until day 21, when the values started to decrease to the lowest values at day 28. Conversely, MTA showed a slight but steady increase in pH until its maximum value at day 21 and then a further decrease at day 28.

**Figure 3:**
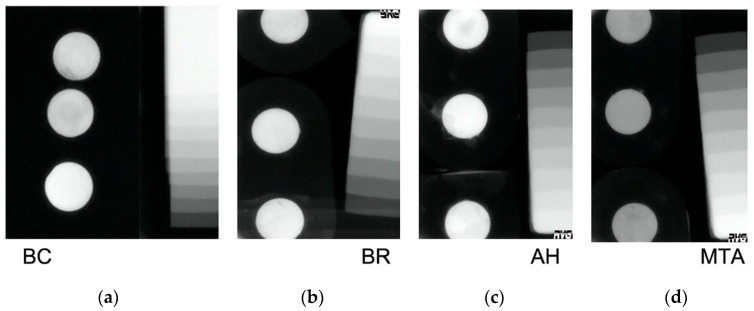
Digital x-rays of (**a**) BC sealer; (**b**) BR sealer; (**c**) AH sealer; (**d**) MTA sealer.

Figure 3 shows digital X-rays of each sealer compared to the Al standard.

**Figure 4:**
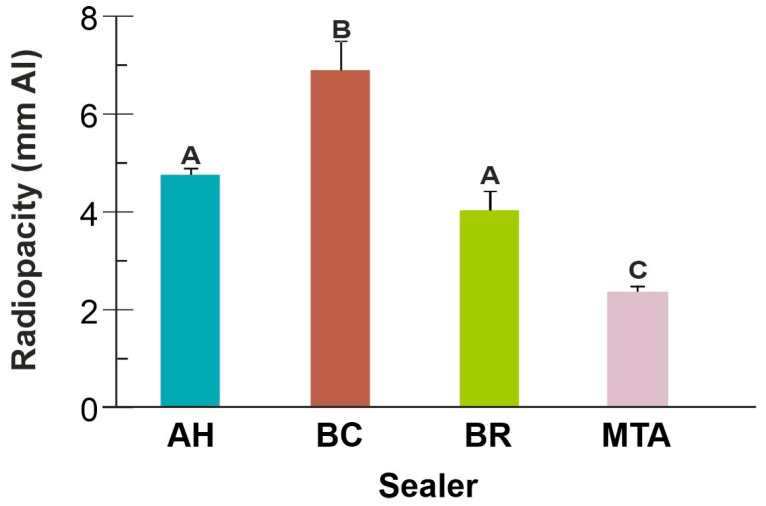
Radiopacity (mean *±* SD) of the tested sealers. Columns sharing the same uppercase letter are not significantly different (*p* > 0.05).

Radiopacity of the tested sealers was in the following order: BC > BR, AH > MTA (*p* < 0.05) (Figure 4).

**Figure 5:**
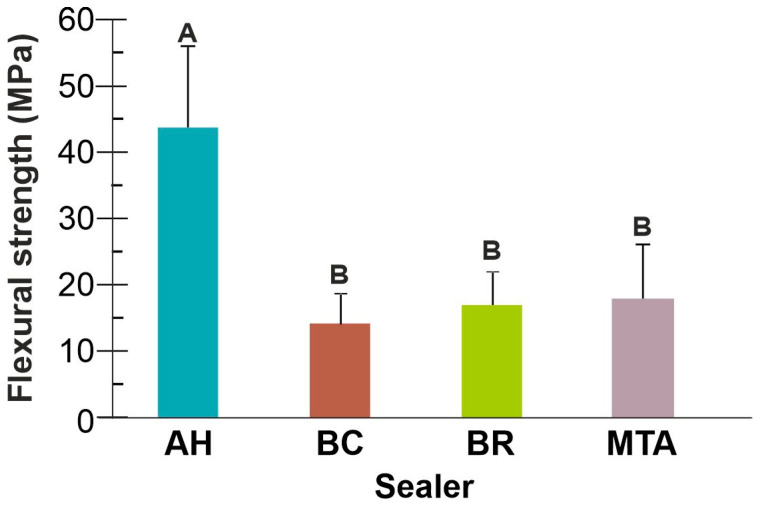
Flexural strength (mean *±* SD) of the tested sealers. Columns sharing the same uppercase letter are not significantly different (*p* > 0.05).

Fs of AH control was significantly higher than that of the three calcium silicate-based sealers (*p* < 0.05). BC, BR and MTA showed no significant differences in Fs values (*p* > 0.05) (Figure 5).

## 4. Discussion

This study showed significant differences between tested sealers in regards to push-out bond strength and pH, radiopacity and Fs. Therefore, the first and the third null hypotheses were rejected. No statistically significant difference in push-out bond strength was found between the disk and conventional method; hence, the second hypothesis was upheld.

It is widely understood that the three-dimensional sealing ability of root canal sealers is of paramount importance for preventing apical and coronal leakage and long-term success of endodontic treatment. Both push-out testing setups, the conventional and disk methods, produced significantly higher bond strength values for resin-based AH control than calcium silicate-based sealers. Greater dislocation resistance of AH may be explained by its chemical composition, i.e., the formation of covalent bonds between epoxy rings in AH and amino groups in dentine collagen [27], low polymerization shrinkage [28] as well as cohesion between the molecules of the sealer [29]. On the other hand, calcium silicate-based sealers bond to dentine by a process known as alkaline etching. Alkalinity of the sealer induces a mineral infiltration zone formation at the interface of the dentine in contact with the sealer [30]. Lower Fs and push-out bond strength values observed for calcium silicate-based sealers than AH control pose a question as to the ability of these sealers to withstand forces generated during post-space preparation or post-cementation. It is currently unknown whether and to what extent the sealer would be able to resist dislocation and/or maintain structural integrity, and this opens an area for further investigation.

MTA sealer exhibited significantly lower bond strength values than the other two calcium silicate-based sealers, BC and BR. This finding is in line with previous studies reporting lower bond strength values of MTA than other sealers [5,10]. Inferior bonding efficiency of MTA could be due to reduced bioactivity, more specifically apatite formation, which was not only quantitatively lower but also delayed in MTA compared to another calcium silicate-based sealer [31]. Reduced apatite formation, especially in interfacial spaces, could not improve bonding efficiency or dislocation resistance, resulting in lower bond strength values compared to other sealers from the same group.

Superior bond strength of BC and BR sealers compared to MTA could be ascribed to interfacial apatite formation as the main adhesion mechanism of calcium silicate-based sealers. Calcium ions released from these sealers interact with phosphate ions from tissue fluids, forming an apatite layer that may reduce external porosity and allow intratubular apatite deposition [32], thus improving bonding efficiency.

All tested sealers showed comparable bond strengths in different locations of the root canal. Previous studies show conflicting evidence, with one being in line with the present finding [33] and another reporting higher bond strength values for AH in the apical region compared to middle and coronal regions [7]. This finding was related to methodological differences, namely the presence of gutta-percha, whose lateral compaction could have resulted in higher apical friction of the obturation material. Bearing in mind that the root canal filling consists of a core material and a sealer, there are at least two interfaces that could be pushed out from the canal space, which could create a systematic source of error, so gutta-percha was not used in the present study setup [34]. Lower values of AH in the apical region could be due to irregular dentine structure, reduced number of dentinal tubules and subsequent inferior sealer infiltration as opposed to coronal and middle regions.

The disk method proved to be comparable to the conventional method for push-out bond strength testing of calcium silicate-based sealers. Additionally, the disk method showed more consistent results, indicating its superiority over the conventional method. It is noteworthy that the disk method requires fewer teeth than the conventional method, simplifying experimental work. A limitation of the disk method is the restricted number of sealers that may be tested due the dimensions of root dentine disks.

The pH of the tested calcium silicate-based sealers was in the range of ~9.5–11.5, with the highest values determined for BC. BR showed comparable results to BC in the present study but with the highest values over shorter periods. The literature shows conflicting data on BR sealer, with one study [13] in accord with the present results, whilst others showed consistently high pH of BR over 6 months [15]. These differences may be related to the powder/liquid composition of BR, requiring manual dosage and mixing. Operator variability in manual handling affects sealer properties, leading to variable alkalinity reported in different studies. MTA showed lower pH than BC and BR, likely due to the lower ion release from the salicylate resin matrix and absence of hydration [19]. Lower pH values for MTA are in accordance with previous studies [13,15], while high alkaline values of BC were comparable to TotalFill BC, which is a sealer of the same chemical composition [14].

As expected, all calcium silicate-based sealers produced higher alkalinity than the control resin-based AH Plus. This is related to composition, hydration but also greater initial solubility and porosity of these sealers in relation to AH [20]. Hydration of calcium silicate-based sealers results in the formation of calcium hydroxide, which, in the presence of water or tissue fluid, subsequently leads to the release of hydroxide and calcium ions, resulting in alkaline pH [22]. Differences in alkalinity within calcium silicate-based sealer are ascribed to size, density and distribution of mineral particles and characteristics of the hydrate phase.

Radiopacity of BC and BR calcium silicate-based sealers determined in the present study was above the threshold of 3 mm Al required by the ISO standard [25]. BC sealer showed substantially higher radiopacity (7 mm Al), more than twice the standard threshold value, exceeding the AH control (4.5 mm Al). These findings are in line with those previously reported by Janini et al. (7.5 mm Al) [35]. Conversely, MTA Fillapex showed radiopacity below the required standard, which was in line with a recent study (2.7 mm Al) [36]. Differences in radiopacity are due to the type and amount of radiopacifying agent in sealer composition [22]. MTA contains calcium tungstate as a radiopacifier, while BR and BC have zirconium oxide. AH contains both of these radiopacifiers, which may contribute to higher radiopacity of this sealer [22]. However, manufacturers do not disclose the amount of radiopacifier in their sealer composition, making the comparison impossible. High radiopacity was also reported for TotalFill BC in another study (6.1 mm Al) [14], albeit there are also some contradictory reports [22,23,24,36,37], which could be due to different measurement techniques. BR demonstrated a radiopacity around 5 mm Al, which is in line with results reported by Siboni et al. (5.2 mm), while Prüllage et al. reported somewhat higher values (6.85 mm Al) [20,21]. High radiopacity is not necessarily desirable, as it may mask internal sealer porosity.

The MTA sealer used in the present study was the “second” generation of this sealer, containing calcium tungstate instead of bismuth oxide. It was previously shown that calcium tungstate has lower radiopacity potential than bismuth oxide and zirconium oxide [38], which could be the reason for the inferior radiopacity of MTA sealer reported in the present study.

Calcium silicate-based sealers exhibited lower Fs than resin-based AH control. This difference can be ascribed to the resin matrix in AH exhibiting greater mechanical resistance than calcium silicate-based sealers. MTA showed somewhat higher Fs values than other calcium silicate-based sealers, albeit with no statistical significance. This could also be due to the presence of salicylate resin in MTA.

As the literature lacks data on Fs of calcium silicate-based sealers, no comparisons can be made. A recent study showed a slightly higher percentage of porosity of MTA (around 6%), compared to around 3.5% of BC and BR, but a greater percentage of larger pores was found in BC followed by BR, whilst MTA had the lowest percentage of the largest-sized pores [39]. This is consistent with Fs values in the present study, being somewhat lower in BC, and higher in BR and MTA, suggesting an effect of the size of sealer porosity on mechanical properties.

In general, the limitations of this in vitro study stem from a laboratory setup that cannot fully replicate the clinical environment, as it lacks the dynamic conditions present in the oral medium. For instance, the moisture for bonding was artificially provided, which does not fully reflect the clinical scenario, but gives a basic idea of the behavior of these materials. In vitro studies often have shorter observation periods compared to long-term clinical scenarios, which can restrict our understanding of how sealers perform over time, including their degradation and interactions with surrounding tissues. Additionally, the use of various methodologies (conventional or digital radiography, variations in sample size and specimen preparation, difference in observation periods, etc.) in in vitro experiments may result in inconsistent outcomes [20,36,37,40,41,42], making it challenging to compare findings across different studies. Calcium silicate-based sealers are highly influenced by various factors related to preparation techniques, operator experience, and environmental conditions. These factors can introduce significant variability, potentially skewing the results and making it difficult to generalize findings to clinical scenarios. Therefore, it is important to standardize protocols, ensure operator consistency, and control environmental conditions as much as possible in order to minimize the impact of these variables on the study’s outcomes. Also, the results derived from a limited selection of examined calcium silicate-based sealers may not accurately reflect the wider range of products available on the market. Understanding these limitations is crucial for interpreting the results obtained in different studies and for making informed decisions regarding the clinical application of calcium silicate-based sealers.

The results of the presented laboratory study should serve as a part of a database that forms the foundation for future clinical research. It is important that upcoming studies utilize standardized methodologies and adhere to ISO recommendations to enable the replication of studies and comparison of experimental results. Future guidelines for testing calcium silicate-based sealers could focus on improving the mechanical properties of these materials while preserving their beneficial biological characteristics. Combining in vitro findings with in vivo studies and clinical trials is essential to gain a complete understanding of sealers’ performance. Since the investigated materials are already widely used in clinical practice, it is of special interest to more profoundly examine their biological properties. In this context, studies are already being undertaken in our group in order to determine the molecular mechanisms underlying material/cell interaction, i.e., potential impact on cell cycle, oxidative stress parameters, mitochondrial function, etc. In addition, long-term clinical studies that analyze the successrate of commercially available calcium silicate-based sealers are needed. In future research, the focus should furthermore be on long-term studies that will address sealer durability and the impact of different clinical conditions on sealer performance.

In vitro studies examining the physicochemical properties of calcium silicate-based sealers carry important clinical implications guiding material selection and facilitating clinicians to choose the most suitable option for optimal sealing and biocompatibility. Moreover, insights into the mechanical strength and stability of the sealers inform clinicians about their longevity and durability within the root canal system. In our study, the control AH sealer showed superior mechanical properties to calcium silicate-based sealers. Translating these finding into clinical conditions would be that these sealers could be more suitable for retreatment [43]. On the other hand, inferior mechanical properties could play a negative role in reinforcing the obturated root canal and the tooth [44]. Calcium silicate-based sealers demonstrated alkalization ability, which, alongside calcium hydroxide formation, makes them suitable for use in cases of external inflammatory root resorptions. These sealers might be preferable for use in situations with middle and apical root canal perforations as well, allowing obturation of the main and perforated canal at the same time [45]. All the factors discussed assist clinicians in choosing the appropriate sealers, resulting in improved outcomes in root canal treatments.

## 5. Conclusions

- Push-out bond strength of calcium silicate-based sealers was lower than that of AH Plus, EndoSequence BC and BioRoot RCS showing superior results compared to MTA Fillapex.
- Push-out bond strength values measured using different testing approaches, the conventional and disk methods, exhibited similar results.
- Since the disk method showed more homogeneous results and required a smaller number of teeth than the conventional method, it may be the preferred method to test the push-out bond strength of calcium silicate-based sealers.
- All calcium silicate-based sealers exhibited higher pH than resin-based AH Plus control sealer.
- Endosequence BC exhibited the highest radiopacity of the tested sealers.
- Radiopacity of MTA Fillapex was the lowest among the tested calcium silicate-based sealers and below the ISO 6876:2001 standard of 3 mm Al.
- The flexural strength of calcium silicate-based sealers was comparable within this group but significantly lower than resin-based AH Plus control.

## Figures and Tables

**Table 1:** Materials used in the present study, sealers, manufacturers, formulation and composition.

Sealer	Manufacturer	Lot Number	Composition *	Mixing
BioRoot RCS (BR)	Septodont, Saint Maur Des Fosses, France	B24408	Powder: tricalcium silicate, zirconium oxide, excipients Liquid: aqueous solution of calcium chloride, excipients	Mix 1 spoon of powder with 5 drops of liquid using a plastic spatula on a paper pad
EndoSequence BC (BC)	Brassler, Savannah, GA, USA	24003SP	Zirconium oxide, calcium silicates, calcium phosphate monobasic, calcium hydroxide, filler, thickening agents	Extrude desired amount using a disposable applicator tip
MTA Fillapex (MTA)	Angelus, Londrina, PR, Brasil	71694	Salicylate resin, diluting resin, natural resin, calcium tungstate, nanoparticulate silica, MTA, pigments	Dual syringe: The dual syringe ensures equal volume mixing in a 1:1 ratio. Press plunger to extrude material directly
AH Plus (AH)	Dentsply DeTrey, Konstanz, Germany	2307000242	Paste A: bisphenol A epoxy resin, bisphenol F epoxy resin, calcium tungstate, zirconium oxide, silica, iron oxide pigments Paste B: dibenzylamine, aminoadamantane, tricyclodecane-diamide, calcium tungstate, zirconium oxide, silica, silicone oil	Extrude equal volume units (1:1). Mix with a metal spatula on a glass mixing pad until achieving homogeneous consistency

* According to manufacturers’ technical data.

**Table 2:** pH values of the tested sealers in different time intervals.

Time, Day	pH (Mean ± SD)			
	Sealer			
	AH	BC	BR	MTA
1	8.79 ± 0.34 A	10.65 ± 0.40 AB	11.08 ± 0.02 A	9.26 ± 0.13 B
3	9.01 ± 0.21 A	11.64 ± 0.17 A	10.63 ± 0.23 A	9.82 ± 0.22 AB
7	9.32 ± 1.08 A	10.88 ± 0.33 AB	10.37 ± 0.22 AB	9.87 ± 0.74 AB
14	9.12 ± 0.60 A	11.74 ± 0.41 A	10.39 ± 0.38 AB	9.98 ± 0.46 AB
21	9.57 ± 0.94 A	11.58 ± 0.48 AB	10.79 ± 0.22 A	11.2 ± 0.99 A
28 days	9.13 ± 0.08 A	9.83 ± 1.19 B	9.67 ± 0.66 B	9.94 ± 0.15 AB

Within each sealer, cells with the same uppercase letter are not significantly different (*p* > 0.05).

## Data Availability

The raw data supporting the conclusions of this article will be made available by the authors on request.
